# Radiogenomics in Clear Cell Renal Cell Carcinoma: A Review of the Current Status and Future Directions

**DOI:** 10.3390/cancers14092085

**Published:** 2022-04-22

**Authors:** Sari Khaleel, Andrew Katims, Shivaram Cumarasamy, Shoshana Rosenzweig, Kyrollis Attalla, A Ari Hakimi, Reza Mehrazin

**Affiliations:** 1 Memorial Sloan Kettering Cancer Center, Department of Urology, New York, NY 10065, USA; khaleels@mskcc.org (S.K.); hakimia@mskcc.org (A.A.H.); 2 Department of Urology, Icahn School of Medicine at Mount Sinai, New York, NY 10029, USA; andrew.katims@mountsinai.org (A.K.); shivaram.cumarasamy@mountsinai.org (S.C.); shoshana.rosenzweig@icahn.mssm.edu (S.R.); kyrollis.attalla@mountsinai.org (K.A.)

**Keywords:** radiogenomics, translational, clear cell renal cell carcinoma

## Abstract

**Simple Summary:**

Clear renal cell carcinoma (ccRCC) is the most common type of renal cancer. As with other malignancies, knowledge of the genetic makeup of ccRCC tumors may provide insights for tumor management and outcomes. However, this normally requires obtaining tissue specimens from the tumor by invasive interventions—surgery or biopsy. Radiogenomics is a field that aims to non-invasively predict the genetic makeup of the tumor based on the tumor’s appearance on conventional imaging, such as CT scans. To achieve this, radiogenomics uses complex machine learning (artificial intelligence) algorithms to process imaging data and build predictive models that can infer a tumor’s genetic makeup and clinical outcomes from its features on conventional imaging. In this article, we searched scientific literature databases for radiogenomic studies in ccRCC, offering a review and critical analysis of these studies. More research and validation are needed before applying radiogenomics in clinical practice.

**Abstract:**

Radiogenomics is a field of translational radiology that aims to associate a disease’s radiologic phenotype with its underlying genotype, thus offering a novel class of non-invasive biomarkers with diagnostic, prognostic, and therapeutic potential. We herein review current radiogenomics literature in clear cell renal cell carcinoma (ccRCC), the most common renal malignancy. A literature review was performed by querying PubMed, Medline, Cochrane Library, Google Scholar, and Web of Science databases, identifying all relevant articles using the following search terms: “radiogenomics”, “renal cell carcinoma”, and “clear cell renal cell carcinoma”. Articles included were limited to the English language and published between 2009–2021. Of 141 retrieved articles, 16 fit our inclusion criteria. Most studies used computed tomography (CT) images from open-source and institutional databases to extract radiomic features that were then modeled against common genomic mutations in ccRCC using a variety of machine learning algorithms. In more recent studies, we noted a shift towards the prediction of transcriptomic and/or epigenetic disease profiles, as well as downstream clinical outcomes. Radiogenomics offers a platform for the development of non-invasive biomarkers for ccRCC, with promising results in small-scale retrospective studies. However, more research is needed to identify and validate robust radiogenomic biomarkers before integration into clinical practice.

## 1. Introduction

Renal cell carcinoma (RCC) is the most common malignant kidney tumor, accounting for approximately 85% of cases [1]. Clear cell carcinoma (ccRCC) is the most common histologic RCC subtype, particularly in advanced RCC (approximately 60–70%, and 90%, respectively) [2]. With increased use of computed tomography (CT) and magnetic resonance-guided imaging (MRI), the incidence of RCC is rising in developed countries, usually at the clinically localized stage [3]. Despite the advancements in cross-sectional imaging technology, their ability to differentiate RCC subtypes and their underlying molecular profiles remain limited [4,5]. 

One approach to improve the diagnostic ability of conventional imaging has been the adoption of advanced computational and statistical methods to process high throughput radiologic features extracted from conventional imaging, giving rise to the field of radiomics [6]. In parallel, our understanding of the genomic profiles of cancers and their potential as diagnostic, prognostic, and therapeutic biomarkers has been advanced by the application of complex computational and statistical methods to analyze high-throughput next-generation sequencing data, allowing for complex genomic, transcriptomic, and epigenomic analyses of tumor specimens. Such analyses in the field of RCC have revealed that in addition to histologic variance, RCC is a genetically diverse disease, with distinct molecular genomic and transcriptomic profiles that correlate with clinical outcomes such as recurrence, progression, and response to systemic therapies [7,8,9,10]. 

Despite the above advances in molecular and radiologic profiling of RCC in general and ccRCC in particular, the current prognostic models remain based on clinical, pathologic, and laboratory characteristics, with the pathologic stage heavily influencing cancer-specific survival [11,12,13,14]. The reliance of these models on pathologic staging makes them inherently invasive, requiring tissue diagnosis based on surgical extirpation or tissue biopsy, with no standardized non-invasive or pre-treatment biomarkers that can be used to classify RCC or predict tumor behavior. This limitation applies to genomic profiling tools, as well, as they also require tissue extraction for their analyses, along with complex and cost-prohibitive translational infrastructures that currently limit their applicability in clinical practice. 

Radiogenomics is a novel field that circumvents the above challenges by utilizing computational machine learning algorithms to correlate radiomic features of disease (radiologic phenotype) with its underlying molecular profile (genotype), thereby offering a platform for the development of non-invasive biomarkers to aid in treatment decisions and disease [15,16]. Of note, while the term “radiogenomics” has been used interchangeably with “radiomics” in literature to describe the study of radiologic features of predictive treatment outcomes, “radiogenomics” is more commonly used to describe the study of the molecular changes underlying the radiologic phenotype of a disease process, including genetic mutations, gene expression, and methylation (epigenetic) changes [15,16,17,18,19]. 

Here, we present an in-depth review of the current state of radiogenomics in ccRCC, and examine the variety of innovative computational models that have been developed in this field to infer the molecular profile of ccRCC from its radiologic phenotype, concluding with a discussion of the field’s current limitations and future directions. 

## 2. Methods

A literature review was performed by querying the PubMed, Medline, Cochrane Library, Google Scholar, and Web of Science databases. We attempted to identify all articles pertaining to radiogenomics and ccRCC. The search terms included “radiogenomics and…” one of the following MeSH search terms: “renal cell carcinoma”, “clear cell renal cell carcinoma”, “kidney cancer”, or “renal cancer”. Titles and abstracts of the articles retrieved from the above search were then screened for relevance. Inclusion criteria were (1) publication in the English language, (2) publication between 1/2009 and 9/2021, (3) and article topic pertaining to radiogenomics of ccRCC. Exclusion criteria included (1) publication before 1/2009, (2) not published in the English language, (3) study topics not pertaining to ccRCC or radiogenomics, (4) duplicate articles, and (5) non-primary literature, e.g., abstracts, review articles, and letters to the editor, which were excluded after being reviewed to identify any missed primary studies.

## 3. Results

**Figure 1:**
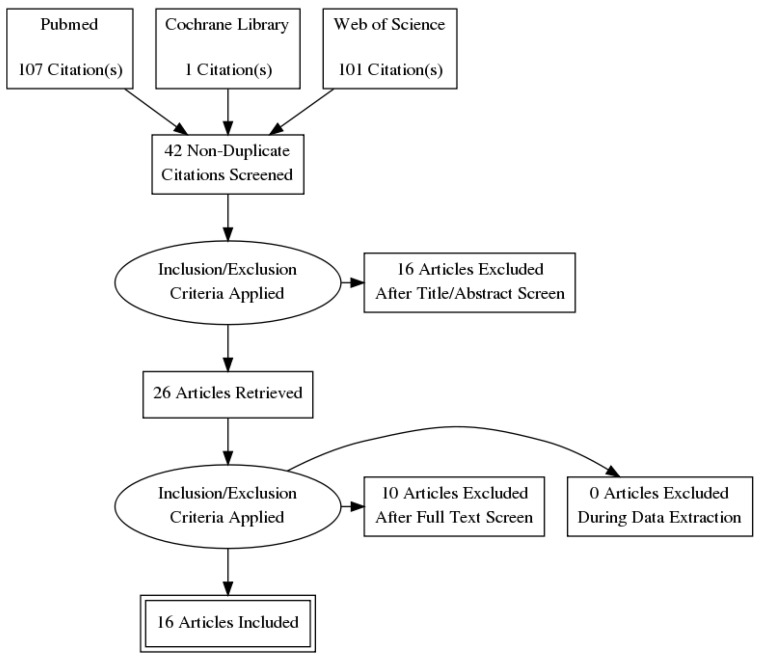
PRISMA flow diagram of article selection criteria.

Overall, 141 articles were identified in our initial search, of which 16 fit our inclusion criteria described above. Radiogenomic features related to mutational status were the most commonly described and targeted features for modeling (eight articles), followed by gene expression (five articles) and epigenetic features (one article). Only two articles developed clinical prognostic models utilizing radiogenomic data. Most articles focused on multiphasic, contrast-enhanced CT scan as the modality of choice, with two paper(s) discussing MRI features. A PRISMA flow chart of our search with inclusion and exclusion criteria can be seen in Figure 1. A list of the included articles along with a summary of their methodology and targeted predictive outcomes can be found in Table 1.

### 3.1. Key Genetic Mutations in ccRCC

Key gene mutations identified in ccRCC include *VHL*, *PBRM1*, *BAP1*, *SETD2*, and *KDM5C*; most of which are located on the short arm of chromosome 3 [35]. Key genetic mutations and their radiogenomic characteristics as well as prognostic value are discussed below, and are summarized in Table 2.

#### 3.1.1. *VHL*

*VHL* gene alteration is the most common mutation in solid ccRCC, with very high frequency (>90%) of biallelic inactivation due to deletion, mutation, or loss of heterozygosity [36,37]. As normal *VHL* protein complexes with other proteins to degrade hypoxia-inducible factor (HIF), *VHL* loss or mutation results in constitutive activation of HIF, promoting cell growth and neo-angiogenesis through the VEGF pathway [38]. Despite its prevalence in ccRCC, the presence of a *VHL* mutation in patients with ccRCC has no prognostic value [10,36,39,40].

#### 3.1.2. *PBRM1*

*PBRM1* is the second most commonly mutated tumor suppressor gene in ccRCC (40–50%), and is often co-deleted with *VHL*. This gene encodes for a nucleosome remodeling complex which limits DNA accessibility to RNA polymerase and transcription factors [35,41]. The prognostic value of *PBRM1* mutation is unclear, with a recent meta-analysis suggesting that mutation and/or loss of in *PBRM1* is a poor prognostic factor in localized disease and a good prognostic factor in advanced disease [42,43]. Other analyses suggest that *PBRM1* mutation status may be predictive of response to immune checkpoint inhibitors [44,45]. *PBRM1* mutations are most associated with solid ccRCC on imaging [20,21].

#### 3.1.3. *BAP1*

*BAP1* gene, present on the short arm of chromosome 3, is mutated in 10–15% of ccRCC, and is typically mutually exclusive of *PBRM1* mutation [35,46]. This tumor suppressor gene encodes a ubiquitin carboxyl-terminal hydrolase that regulates with downstream targets involved in cell breakdown and replication, with *BAP1* inactivation resulting in uncontrolled cell proliferation [41,47]. *BAP1* mutation has been associated with more aggressive disease and lower overall survival in ccRCC, with coagulative necrosis and high Furman grade on tumor pathology [48,49].

Typical radiologic features associated with *BAP1* mutation include renal vein invasion, ill-defined tumor margins, and intratumor calcifications. Of note, *BAP1* mutations were absent in multicystic ccRCC [20,21].

#### 3.1.4. *SETD2*

As with *BAP1*, *SETD2* is a tumor suppressor gene located on the short arm of chromosome 3, and is mutated in approximately 10–15% of ccRCC [35]. *SETD2* loss has been associated with poor prognosis in nonmetastatic ccRCC [48]. Radiomic analyses note *SETD2* mutation to be absent in multicystic ccRCC, with no consistent CT imaging findings predictive of *SETD2* mutation in solid ccRCC [20,21].

#### 3.1.5. *KDM5C*

*KDM5C* is mutated in approximately 6–7% of ccRCC [35]. The prognostic value of *KDM5C* remains debated, with one series noting an association with prolonged survival in metastatic ccRCC [50]. Tumors with *KDM5C* mutation were consistently associated with renal vein invasion on CT and absent in multicystic ccRCC [20,21].

### 3.2. Overview of Radiogenomics Workflow 

**Figure 2:**
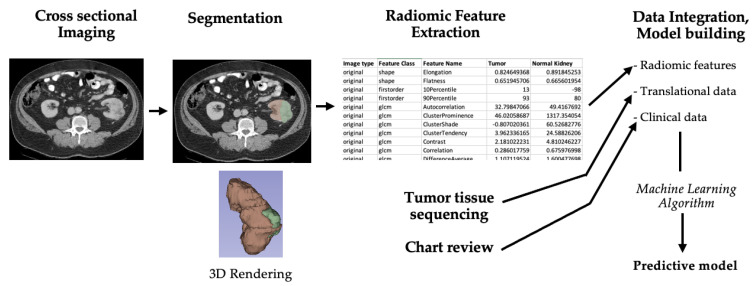
Flowchart showing typical radiogenomic workflow. Using cross-sectional images, a region of interest (ROI) that contains either the whole tumor or subregions within the tumor can be identified and outlined using manual in process called segmentation, using semi-automated, or automated segmentation software. Some segmentation software, such as 3D Slicer (shown above) allow for further ROI rendering in 3D dimensions. Quantitative radiomic features are extracted from ROI using separate or built-in radiomic feature extraction modules. Finally, this data is integrated with corresponding tumor molecular profile, as well as patient clinical data. These data are then processed using machine learning algorithms to develop diagnostic, predictive, or prognostic models for outcomes of interest.

As mentioned earlier, radiomics refers to the extraction and analysis of quantitative imaging features from cross-sectional imaging modalities, while radiogenomics refers to the study of the translational phenotype underlying these imaging features [51]. A typical radiogenomic workflow is shown in Figure 2. First, the region of interest (ROI), being the tumor and/or specific tumor sub-region(s), is “segmented”, i.e., outlined in all slices of the imaging study using manual or semi-automated segmentation software, generating a 3D rendering of the ROI. Next, specialized software is used to extract hundreds to thousands of radiomic features from the ROI “agnostically”, with no knowledge of its clinical context or molecular profile, such as malignant/benign status, RCC subtype, or mutational profile. Extracted features may include first-order statistics of voxel intensity and distribution, as well as higher level metrics of tumor shape, texture, and 2D/3D features, extracted from one or more phases of the imaging study. Next, machine learning (ML) algorithms are used to process these raw features to identify the subset of features that are predictive of an outcome of interest, which in radiogenomics would include specific gene mutation, gene expression profile, or clinical outcome [52]. The radiogenomic model constructed from this subset of features is usually “trained” using one dataset, followed by external cross-validation in an independent dataset. 

### 3.3. Mutational Radiogenomic Biomarkers

In this section, we review articles that develop radiogenomic models to predict tumor gene mutational profile in ccRCC, which mostly focused on the previously discussed *PBRM1* and *BAP1* mutations. 

Chen et al. (2018) presented a radiogenomic predictive model to predict multiple ccRCC gene mutations (*VHL*, *PBRM1*, and *BAP1*) using quantitative CT features. To achieve this, they developed a new multi-classifier multi-objective (MCMO) model to train their model against multiple objectives (ccRCC mutations of interest) rather than a single objective. After training their model using an institutional cohort of 57 patients, it was validated using The Cancer Genome Atlas’s Kidney-Renal Cell Carcinoma (TCGA-KIRC) cohort. Their model achieved prediction accuracy of 0.81, 0.78, and 0.90 for *VHL*, *PBRM1*, and *BAP1* genes, respectively, with AUC ≥ 0.86, sensitivity ≥ 0.75, and specificity ≥ 0.80 [22].

Focusing on *PBRM1* mutation, which is a likely good prognostic factor in advanced ccRCC [42,53], Kocack et al. (2019) developed two predictive radiogenomic models using artificial neural network algorithm (ANN) and RF algorithms to differentiate ccRCC tumors by *PBRM1* mutations status in the TCGA-KIRC cohort (45 patients; 29 without and 16 with *PBRM1* mutation). Their ANN model demonstrated an accuracy of 88.2% (AUC = 0.925) compared to 95.0% (AUC = 0.987) with the RF algorithm. However, they did not directly evaluate their model’s correlation with clinical outcomes [24]. 

The same group (Kocak et al., 2020) then developed an RF-based radiogenomic model for the prediction of *BAP1* mutation status, which carries poor prognostic implications in ccRCC [20,21,25], in a subset of 65 patients from TCGA-KIRC (13 with and 52 without *BAP1* mutation). This model correctly classified *BAP1* mutation status in 84.6% of cases (AUC = 0.897) [20,21,25]. The same algorithm (RF) and dataset (TCGA-KIRC) were used by Feng et al. (2020) to also predict *BAP1* mutation status, but using different segmentation and radiomic feature extraction platforms; the model accurately classified 83% (AUC = 0.77) of *BAP1* mutation status with a sensitivity of 0.72, specificity of 0.87, and precision of 0.65 [26]. Finally, targeting *BAP1* status as well, Ghosh et al. (2015) developed four imaging phase-specific *BAP1* classifiers for the non-contrast, cortico-medullary, nephrographic, and excretory phases of CT studies from the TCGA-KIRC cohort (78 patients). Interestingly, their model utilized 3D feature extraction to evaluate intra-tumoral heterogeneity (ITH), which they hypothesized reflected *BAP1* mutational status [27]. In contrast, none of the previously discussed studies considered ITH in their model design, despite its known prevalence and influence on clinicopathological and molecular assessment of ccRCC, as one tumor area’s molecular profile may be different from another’s, with downstream implications for the extracted radiomic features in these models [54,55].

### 3.4. Beyond Gene Mutations: Transcriptomic and Epigenetic Radiogenomic Biomarkers

As discussed earlier, the clinical relevance of some of the most common mutations in ccRCC remains unclear, particularly given the low prevalence of some of these mutations, limiting their potential as clinical biomarkers [20,21,35,36,37,38,39,41,42,43,44,45,46,47,48,49,50,56]. In contrast, transcriptional (gene-expression based) signatures have been shown to be better tools for classifying ccRCC into clinically-relevant molecular subtypes [57,58]. Such subgrouping classifications include clear cell type A (ccA) and clear cell type B (ccB) described by Brannon et al. using microarray data. Using these tumor classifications, they noted a prognostic difference between the two groups; ccA was significantly associated with better survival compared to ccB [8]. As transcriptomic research shifted from microarray to next-generation RNA-sequencing (RNA-Seq) technology, Brooks et al. developed a 34-gene expression signature, ClearCode34, for the classification of localized ccRCC tumors to ccA and ccB categories using RNA-Seq data [57]. Another attempt at transcriptomic profiling of ccRCC performed by the TCGA group using unsupervised clustering of RNA-Seq data identified four subgroups, m1–m4. Supervised clustering of these subgroups against ccA/ccB subgrouping noted cluster m1 to correspond to ccA, m2–m3 to correspond to cluster ccB, and m4 to correspond to the 15% of tumors that did not align with either ccA or ccB. As with ccA, the m1 subgroup had a survival advantage over m2–m4, sharing some of its genes with the *PBRM1* mutation group and functions within the chromatin remodeling process. In contrast, the m3 subgroup harbored mutations of *PTEN* and *CDKN2A*, while patients within the m4 subgroup exhibited a higher frequency of *BAP1* mutations [35]. Furthermore, these subtypes were associated with distinct radiomic features; the m1 subgroup had well-defined tumor margins (vs. ill-defined, OR = 2.104; CI 1.024–4.322), while the m3 subtype was less frequently associated with well-defined tumor margins (OR = 0.421; CI 0.212–0.834) and had more collecting system invasion (OR = 2.164; CI 1.090–4.294) and renal vein invasion (OR 2.120; CI 1.078–4.168). There were no significant CT findings with the m2 or m4 subgroups [7]. 

In this section, we explore radiogenomic models that correlate radiologic tumor “phenotype” to its underlying transcriptomic and epigenetic molecular profile, rather than genetic mutational profile. In addition to their ability to reflect variations in individual tumor gene expression and hypermethylation patterns, these models are potentially more applicable to clinical practice than radiogenomic models that predict only genomic mutational profile, given the ability of their targeted molecular expression profiles to better reflect survival and therapeutic outcomes [29,30]. 

#### 3.4.1. Transcriptomic Radiogenomic Biomarkers

Using the aforementioned transcriptomic ccA/ccB ccRCC subtype, Yin et al. developed a model utilizing radiomic features extracted from MRI/PET data to classify ccRCC into ccA or ccB subtypes, using sparse partial least squares discriminant analysis (SPLS-DA) to build two predictive models—one with the radiomic features alone, and one incorporating clinical characteristics, mRNA, microvascular density, and molecular subtype information. The correct classification rate was 87% vs. 95.6% using the radiomic signature alone vs. the combined signature, respectively [29]. However, the study utilized a small cohort (23 specimens from eight primary ccRCC patients), and PET imaging is not usually used for evaluation nor surveillance of localized ccRCC. 

#### 3.4.2. Epigenetic Radiogenomic Biomarkers

At the epigenetic level, DNA methylation, particularly the runt-related transcription factor 3 (RUNX3) gene, has been correlated with overall survival [30]. Cen et al. (2019) evaluated the correlation between RUNX3 methylation levels and certain imaging features on CT in ccRCC. Among somatic CT findings, margin status (ill vs. well-defined; OR 2.685; CI 1.057–6.820) and intratumoral vascularity (present or absent; OR 3.286; CI 1.367–7.898) were significant independent predictors of high RUNX3 methylation levels on multivariate logistic regression [30].

### 3.5. Beyond Predicting Molecular Profile: Radiogenomic Models as Clinical Biomarkers

While the above reviewed studies present impressive analyses and methods for inferring tumor biology using radiomic features, the clinical relevance of their proposed features and models remains unproven without direct assessment of their ability to predict clinical outcomes. In this section, we review a few notable radiogenomic studies that go beyond correlating only radiomic and molecular features to also demonstrating a direct correlation between their radiogenomic biomarkers with clinical outcomes for ccRCC. 

Focusing on radiologic features predictive of survival outcomes, Huang et al. performed radiogenomic analysis of CT imaging for ccRCC cases with corresponding RNA expression data in the TCGA-KIRC cohort. LASSO-COX regression was used to identify prognostic radiomic features and prognostic gene signatures. An RF algorithm was then used to combine prognostic and radiomic features into a radiogenomic prognostic model. The radiogenomic model outperformed the radiomic features-only model at predicting overall survival at 1, 3, and 5 years (average AUCs for 1-, 3-, and 5-year survival of 0.814 vs. 0.837, 0.74 vs. 0.806, and 0.689 vs. 0.751, respectively) [31].

In another study, Jamshidi et al. constructed a radiogenomic risk score (RSS) using a cohort of patients who underwent nephrectomy with corresponding micro-array-derived gene expression data. Following CT imaging feature extraction, multivariate regression was used to identify features most predictive of variation in supervised principal component (SPC) gene expression analysis. These features were used to constitute their RSS, which was validated in a separate patient cohort (70 for validation of the signature’s correlation with micro-array results, 77 for correlation of signature with disease-free survival). The RRS exhibited a statistically significant correlation with micro-array SPC variation (R = 0.57, *p* < 0.001, classification accuracy 70.1%, *p* < 0.001) and disease-specific survival (log-rank *p* < 0.001), accounting for stage, grade, and performance status (multivariate Cox model *p* < 0.05, log-rank *p* < 0.001) [32]. In a separate study, the RRS was validated in a cohort of 41 mRCC patients undergoing cytoreductive nephrectomy (CRN) and pre-surgical bevacizumab, noting that it was able to stratify radiological progression-free survival (rPFS) in this cohort; patients with a low RSS vs. high RSS had longer rPFS (25 months vs. 6 months; *p*  =  0.005) and OS (37 months vs. 25 months; *p*  =  0.03) [33].

Focusing on micro-RNA (miRNA) expression in RCC, Marigliano et al. (2019) evaluated the correlation between a variety of radiomic features extracted from a cohort of 20 ccRCC patients, and their expression levels of selected microRNAs. Specifically, they examined the correlation of these features with miR-21-5p, miR-210-3p, miR-185-5p, miR-221-3p, and miR-145-5p, which had been shown to correlate with clinical outcomes in ccRCC [59]. They found no significant correlation between their extracted features and expression of any of their evaluated miRNAs [28].

While the molecular profiling of tumors using transcriptomic and epigenetic signatures offers more clinically meaningful biomarkers than genomic mutational signatures, it overlooks the critical role of the tumor’s stromal and immune background, collectively referred to as the tumor microenvironment (TME), in the prognosis and therapeutic response of ccRCC. This role has been increasingly recognized with the rise of immunotherapy (IO) regimens, which target the immune component of the TME as monotherapy or in combination with TKI agents, which target the angiogenic component of the TME, as well [60,61,62,63].

In this regard, Udayakumar et al. (2021) utilized dynamic contrast-enhanced MRI (DCE-MRI) imaging to identify areas of high and low colocalized enhancement within tumor regions of 49 ccRCC patients undergoing DCE-MRI prior to nephrectomy, followed by targeted sampling and RNA-sequencing of nephrectomy specimen regions corresponding to these areas. They found enhancement-high tumors to exhibit upregulated angiogenesis-related TME gene expression signatures, while enhancement-low areas exhibited higher levels of immune (T-cell infiltration) TME signatures, confirmed by immunohistochemical analysis. They then validated their model’s ability to predict response to TKI or immunotherapy (IO) treatments in a cohort of 19 patients with metastatic ccRCC, noting better PFS with TKI in the enhancement-high compared to enhancement-low tumor groups (adjusted *p* < 0.0001), but no significant difference in PFS with IO between the two groups [34].

## 4. Discussion

In this review, we provided an overview of radiogenomic studies in ccRCC, the most common subtype of RCC, and renal malignancies in general. While the majority of studies focused on developing models for the prediction of tumor gene mutational profiles, we noted a shift towards the prediction of gene expression patterns and epigenetic changes within the tumor as well as the tumor microenvironment, which provide better insights into tumor biology and potential therapeutic response than isolated gene mutation profiles. A minority of the reviewed models were also shown to be predictive of relevant clinical outcomes, such as cancer-specific survival and response to systemic therapy in advanced ccRCC. Such models may complement the management of localized renal tumors to confirm whether the tumor exhibits high- or low-risk features that may warrant more aggressive management vs. surveillance, and in advanced ccRCC to determine the optimal systemic treatment regimens based on radiogenomic assessment of the tumor and its microenvironment.

However, the clinical applicability of these models remains limited by several factors. First, all the predictive models presented by the reviewed studies were developed using relatively small cohorts, mostly utilizing the same publicly available cohort (TCGA-KIRC), potentially overfitting their models to this cohort, with only a few performing external validation in independent cohorts. Second, the quality of CT studies is dependent on a variety of technical factors, such as the CT scanner, acquisition mode, and voxel reconstruction algorithms, thereby affecting the quality of extracted radiomic data. Third, the extracted radiomic features come from segmented tumor images, which are usually manually or semi-automatically delineated by a human user—a process that is inherently subjective and liable to inter-observer variability. Fourth, there are no standardized protocols or software tools for radiomic feature extraction, with the concern that the hundreds to thousands of radiomic features extracted by one software package are often redundant and difficult to replicate by other software packages [64], thus limiting the external validity of the models developed from these features. The Image Biomarker Standardization Initiative is a recent attempt at addressing this issue, establishing a standardized set of unique radiomics features [65], although compliance with this initiative has yet to be seen in radiogenomics publications. This lack of a unified radiomic feature extraction protocol or terminology limits our ability to compare the subsets of predictive radiomic features across different models, which consequently limits the ability to identify any consistent radiomic features across different models. Furthermore, it hinders attempts to identify the biologic processes that may underlie changes in these radiomic features. Fifth, most of the models did not consider intra-tumoral heterogeneity, despite its known influence on clinicopathological and molecular assessment of ccRCC, with different tumor regions expressing different pathologic phenotypes and molecular profiles, with implications for therapeutic response. Therefore, a radiogenomic model that was trained to treat the entire tumor region as a single homogenous entity may not accurately predict a tumor’s molecular profile or its correlated clinical outcomes. Finally, while the ultimate measure of any biomarker is to show reliable and independent correlation with clinical outcomes, complementing standard-of-care biomarkers and predictive tools, most of these studies focused on developing models to predict molecular profiles without directly demonstrating clinical relevance as an independent biomarker of key prognostic and therapeutic outcomes, or in combination with established predictive models and nomograms. These are critical limitations that must be addressed for radiogenomics to be reliably used as a tool in clinical practice.

Despite these limitations, the above studies demonstrate the potential of radiogenomics as a non-invasive biomarker of tumor biology, utilizing complex computational tools to identify radiologic tumor features that correlate with genomic, transcriptomic, and/or epigenetic features of the tumor, and their downstream clinical implications.

## 5. Conclusions

The field of radiogenomics is a potentially promising tool in constructing personalized cancer care, offering a novel non-invasive translational biomarker that can be used for molecular profiling of clear cell renal carcinoma. However, this field remains relatively immature, and all the reviewed studies in the field rely on retrospective analyses, with no large-scale prospective trials, a critical requirement for the implementation of this technology in clinical practice.

## Figures and Tables

**Table 1:** Summary of included radiogenomic studies in this review. Studies were selected based on the literature search strategy summarized in the methods section and Figure 1. Most studies utilized studies from the publicly available TCGA-KIRC cohort, specifically focusing on patients in that database with corresponding imaging studies in the TCIA portal.

Author and Year	Imaging Modality	Primary Outcome of Interest	Machine Learning Algorithm	Summary of Results	Notes
Karlo et al. (2014) [20]	Multiphase CT	Investigate association between CT features of ccRCC and mutations in *VHL*, *PBRM1*, *SETD2*, *KDM5C*, or *BAP1*	N/A—Development of a predictive model was not intended	Mutations of *VHL* were significantly associated with well-defined tumor margins, nodular tumor enhancement, and gross appearance of intratumoral vascularity. Mutations of *KDM5C* and *BAP1* were significantly associated with evidence of renal vein invasion. Mutations of *SETD2*, *KDM5C*, and *BAP1* were absent in multicystic clear cell RCC; mutations of *VHL* and *PBRM1* were significantly more common among solid clear cell RCC	Retrospective review of institutional cohort of 233 patients with ccRCC and known mutation status for genes of interest.
Shinagare et al. (2015) [21]	Multiphase CT and MRI	Investigate association between CT/MRI features of ccRCC and mutations in *VHL*, *BAP1*, *PBRM1*, *SETD2*, *KDM5C*, and *MUC4*	N/A—Development of a predictive model was not intended		Retrospective review of 103 patients with CT and/or MRI images; majority (81) were CT-only.
Chen et al. (2018) [22]	Multiphase CT	Create a ML model to differentiate ccRCC tumors by radiomic features reflective of genetic mutation profile (*VHL*, *PBRM1*, *BAP1*)	Multi-classifier multi-objective (MO) and MO optimization algorithm	Model AUC ≥ 0.86, sensitivity ≥ 0.75, and specificity ≥ 0.80	Used a relatively small (57 patients) institutional cohort for training and validation. The model was designed to predict multiple rather than single outcome (mutation).
Li et al. (2019) [23]	Multiphase CT	Create a ML model to differentiate ccRCC from non-ccRCC tumors by radiomic features	Random forest (RF) and minimum redundancy maximum relevance (mRMR)	Model AUC of 0.949 and an accuracy of 92.9% vs. an AUC of 0.851 and an accuracy of 81.2% for the RF and mRMR models, respectively	Used a large (255 patients) institutional cohort for training and validation. Secondary outcome was correlation of predictive features with *VHL* mutational status, with false discovery rate *p*-value < 0.05.
Kocack et al. (2019) [24]	Multiphase CT	Create a ML model to differentiate ccRCC tumors by radiomic features reflective of *PRBM1* mutation status	Artificial neural network (ANN) and RF algorithms	Model accuracy of 88.2% (AUC = 0.925) vs. 95.0% (AUC = 0.987) for the ANN vs. RF models	Used only 45 patient studies from the TCGA-KIRC cohort for training the model (29 *PRBM1*-unmuated, 16 *PRBM1*-mutated).
Kocack et al. (2020) [25]	Multiphase CT	Create a ML model to differentiate ccRCC tumors by radiomic features reflective of *BAP1* mutation status	RF algorithm	Model specificity of 78.8% and precision of 81% for presence and absence of *BAP1* mutations, respectively	Used 65 patients from TCGA-KIRC for training the model (13 with and 52 without *BAP1* mutation).
Feng et al. (2020) [26]	Multiphase CT	Create a ML model to differentiate ccRCC tumors by radiomic features reflective of *BAP1* mutation status	RF algorithm	Model AUC = 0.77, sensitivity of 0.72, specificity of 0.87, and precision of 0.65	Used 56 patients (9 *BAP1*-mutated, 45 *BAP1*-unmutated) TCGA-KIRC for training the model.
Ghosh et al. (2015) [27]	Multiphase CT	Create a ML model to differentiate ccRCC tumors by radiomic features reflective of *BAP1* mutation status	RF algorithm	AUCs of 0.66, 0.62, 0.71, and 0.52 for the non-contrast, cortico-medullary, nephrographic, and excretory phases, respectively	Used TCGA-KIRC for training and validation cohorts (78 patients). Developed separate classifiers for *BAP1* in the non-contrast, cortico-medullary, nephrographic, and excretory phases. Utilized 3D feature extraction to evaluate intra-tumoral heterogeneity.
Bowen et al. (2019) [7]	Multiphase CT	Describe radiomic features associated of molecular TCGA subtypes (m1–m4)	N/A—Development of a predictive model was not intended	The m1 subgroup had well-defined tumor margins (vs. ill-defined, OR = 2.104; CI 1.024–4.322). The m3 subgroup was less frequently associated with well-defined tumor margins (OR = 0.421; CI 0.212–0.834); more collecting system invasion (OR = 2.164; CI 1.090–4.294) and renal vein invasion (OR 2.120; CI 1.078–4.168). There were no significant CT findings with the m2 or m4 subgroups	TCGA cohort was used for this assessment.
Marigliano et al. (2019) [28]	Multiphase CT	Describe radiomic features associated with miRNA expression	N/A—Development of a predictive model was not intended	There were no significantly associated texture-specific features with expression of any of the evaluated miRNAs	Pilot study using small institutional cohort of 20 patients.
Yin et al. (2018) [29]	PET and MRI	Develop a combined PET/MRI model + other features to predict ccRCC molecular subtype (ccA vs. ccB)	ML was not used to build the predictive model	Correct classification rate was 87% vs. 95.6% using the radiomic signature alone vs. the combined signature (radiomic signature + several clinical features)	Very small training/test subset (23 specimens from 8 primary ccRCC patients). Sparse partial least squares discriminant analysis (SPLS-DA) was used to build their predictive models.
Cen et al. (2019) [30]	Multiphase CT	Identify CT imaging features predictive of high *RUNX3* methylation levels	N/A—Development of a predictive model was not intended	Well vs. poorly defined margin status (OR 2.685; CI 1.057–6.820), and present/absent intratumoral vascularity (OR 3.286; CI 1.367–7.898) were all significant independent predictors of high *RUNX3* methylation on multivariate regression	
Huang et al. (2021) [31]	Multiphase CT	Development of a radiogenomic model to predict overall survival in ccRCC using gene expression data	LASSO-COX regression to identify a prognostic radiomic signature, then RF to combine the radiomic and prognostic gene signatures	The radiogenomic model outperformed the radiomic features-only model at predicting overall survival at 1, 3 and 5 years (average AUCs for 1-, 3-, and 5-year survival of 0.814 vs. 0.837, 0.74 vs. 0.806, and 0.689 vs. 0.751, respectively)	Trained model using TCGA-KIRC dataset (205 patients).
Jamshidi et al. (2015) [32]	Multiphase CT	Development of a radiogenomic risk score (RSS) to predict gene expression results from a microarray assay	None—Multivariate regression was used to identify features most predictive of variation in supervised principal component (SPC) gene expression analysis	Significant correlation of RSS with the microarray gene signature (R = 0.57, *p* < 0.001; classification accuracy 70.1%, *p* < 0.001) Significant correlation of RSS with disease-specific survival: log-rank *p* < 0.001	RSS was developed from data in a 70-patient cohort, with validation in a separate cohort (70 for validation of the signature’s correlation with micro-array results, 77 for correlation of signature with disease-free survival).
Jamshidi et al. (2016) [33]	Multiphase CT	Correlation of RSS developed in above study with radiologic progression free survival (rPFS) in a cohort of 41 mRCC patients undergoing CRN and pre-surgical bevacizumab	None—Purpose of study was to compare rPFS in the low- vs. high-RSS cohorts	Patients with a low RSS vs. high RSS had longer rPFS (25 months vs. 6 months; *p* = 0.005) and OS (37 months vs. 25 months; *p* = 0.03)	
Udayakumar et al. (2021) [34]	Dynamic contrast-enhanced MRI	Correlation of enhancement scores for tumors with their TME expression signature	None	Enhancement-high tumors exhibited upregulated angiogenesis-related TME gene signatures, while enhancement-low areas exhibited higher levels of T-cell infiltration signatures. Better PFS with TKI in the enhancement-high compared to enhancement-low tumor groups (adjusted *p* < 0.0001), but no significant difference in PFS with IO between the two groups	Cutoff for determining tumors to have high or low enhancement/angiogenesis/infiltration was relative to the median value of the distribution of these values in the training cohort. Authors did not utilize any previously published TME signatures for angiogenesis or immune infiltration.

**Table 2:** Summary of the top 5 most common gene mutations in ccRCC.

Gene Mutation	Frequency in ccRCC (%)	Protein Function	Clinical and Prognostic Implications	Associated Features on CT Imaging
*VHL*	>90%	Tumor Suppressor	None	Defined tumor margins, nodular tumor enhancement, intratumor vascularity
*PRBM1*	40–50%	Tumor Suppressor	Inconsistent clinical significance in localized ccRCC; may be predictive of better prognosis and response to immune checkpoint inhibitors in metastatic ccRCC	Solid ccRCC
*BAP1*	10–15%	Tumor Suppressor	Poor prognosis	Renal vein invasion, ill-defined tumor margins, and intratumor calcificationsAbsent in multicystic ccRCC
*SET2D*	10–15%	Tumor Suppressor	Poor prognosis	Inconsistent Absent in multicystic ccRCC
*KDM5C*	6–7%	Tumor Suppressor	Good prognosis	Renal vein invasion Absent in multicystic ccRCC
